# Validation of Integrated Periphyton Technology in Mixed Sex Culture of Giant Freshwater Prawn, *Macrobrachium Rosenbergii*: Insights into Impact of Heterogenous Independent Differentiation and Gender on Growth Dynamics in Grow Out

**DOI:** 10.21315/tlsr2025.36.3.3

**Published:** 2025-10-31

**Authors:** David Marioni, Nor Azman Kasan, Liew Hon Jung, Victor Torres Rosas, Ponnumony Vethamony, Jassim Abdulla Al-Khayat, Mhd Ikhwanuddin

**Affiliations:** 1 Higher Institution Centre of Excellence (HICoE), Institute of Tropical Aquaculture and Fisheries, Universiti Malaysia Terengganu, 21300 Kuala Nerus, Terengganu, Malaysia; 2 Department of Aquaculture, Faculty of Fisheries and Marine, Universitas Airlangga, Kampus C Mulyorejo, Surabaya 60115, Indonesia; 3 Qatar University, Environmental Science Center, PO Box 2713, Doha, Qatar

**Keywords:** Integrated Periphyton Technology (IPT), Recirculating Aquaculture System (RAS), Zero-discharge Aquaculture, Grow-out Phase, Morphotype Differentiation, Teknologi Perifiton Bersepadu (IPT), Sistem Akuakultur Kitar Semula (RAS), Akuakultur Tanpa Pelepasan, Fasa Tumbesaran, Pembezaan Morfotip

## Abstract

This study evaluated the performance of mixed sex postlarval (PL) populations of giant freshwater prawn, *Macrobrachium rosenbergii*, cultured under Integrated Periphyton Technology (IPT) and Recirculating Aquaculture System (RAS) conditions. The trials were conducted in triplicate over 44 days of nursery culture (T1), followed by 60 days of growth after size separation (T2 and T3). The carbon-to-nitrogen (C:N) ratios were optimised through molasses supplementation. The water quality parameters in both systems remained within acceptable ranges. Key performance indicators, including body weight, survival rate, average daily gain, specific growth rate, harvest biomass and feed conversion ratio (FCR), were analysed across T1 and T2. While nursery performance (T1) was not significantly different between the IPT and RAS systems, Morphological distinctions among male morphotypes (BC, OC and SM) and females were characterised, with sex-specific growth performance compared across T1 and T2, as were size-separated populations in T3. IPT demonstrated more effective production of Blue Claw (BC) prawns during the grow-out phase (T2). IPT, a zero-discharge system, matched or outperformed RAS, while eliminating the need for external effluent management, while equivalent FCR did not establish periphyton as a supplemental food source within the production volume. Although size separation yielded variable benefits, enhancement of overall productivity was inconclusive. This study highlights IPT as a sustainable alternative to conventional RAS, offering equivalent FCR, and lower energy consumption, and land resource requirement. Further investigations are warranted to optimise intensive IPT systems for economic feasibility and environmental sustainability, contributing to broader advancements in aquaculture.

## Abstract (fr)

**Abstrak:**

Kajian ini menilai prestasi populasi pascalarva (PL) jantina bercampur bagi udang galah, Macrobrachium rosenbergii, yang diternak di bawah keadaan Teknologi Perifiton Bersepadu (IPT) dan Sistem Akuakultur Kitar Semula (RAS). Ujian telah dijalankan secara triplikat selama 44 hari ternakan asuhan (T1), diikuti dengan 60 hari ternakan tumbesaran selepas pengasingan saiz (T2 dan T3). Nisbah karbon kepada nitrogen (C:N) telah dioptimumkan melalui penambahan molases. Parameter kualiti air dalam kedua-dua sistem kekal dalam julat yang boleh diterima. Penunjuk prestasi utama, termasuk berat badan, kadar kehidupan, purata pertumbuhan harian, kadar pertumbuhan khusus, biomas tuaian dan nisbah penukaran makanan (FCR), telah dianalisis merentasi T1 dan T2. Walaupun prestasi ternakan asuhan (T1) tidak menunjukkan perbezaan yang ketara antara sistem IPT dan RAS, perbezaan morfologi antara morfotip jantan (BC, OC dan SM) dan betina telah dicirikan, dengan prestasi pertumbuhan khusus jantina dibandingkan antara T1 dan T2, begitu juga populasi yang diasingkan saiznya dalam T3. IPT menunjukkan hasil pengeluaran udang galah Blue Claw (BC) yang lebih berkesan semasa fasa ternakan tumbesaran (T2). IPT, sebagai sistem tanpa pelepasan, menyamai atau mengatasi RAS, sambil menghapuskan keperluan pengurusan efluen luaran, manakala FCR yang setara tidak membuktikan perifiton sebagai sumber makanan tambahan dalam jumlah hasil pengeluaran. Walaupun pengasingan saiz memberikan manfaat yang berubah-ubah, peningkatan terhadap produktiviti keseluruhan adalah tidak konklusif. Kajian ini menonjolkan IPT sebagai alternatif yang mampan kepada RAS konvensional, dengan menawarkan FCR yang setara, penggunaan tenaga yang lebih rendah, dan keperluan sumber tanah yang lebih sedikit. Penyelidikan lanjut adalah diperlukan untuk mengoptimumkan sistem IPT intensif bagi daya maju ekonomi dan kelestarian alam sekitar, sekali gus menyumbang kepada kemajuan lebih luas dalam bidang akuakultur.

**HIGHLIGHTS**

- Mixed sex postlarval (PL) populations of giant freshwater prawn, *Macrobrachium rosenbergii*, were cultured under Integrated Periphyton Technology (IPT) and Recirculating Aquaculture System (RAS) for 44 and 104 days.
- Morphological distinctions among male morphotypes (BC, OC and SM) and females were characterised, with growth performance compared.
- Trials demonstrated IPT to be a sustainable alternative to conventional RAS, offering equivalent FCR and both lower energy consumption and land resource requirement.

## INTRODUCTION

Integrated Periphyton Technology (IPT) is in the class of systems that includes Biofloc Technology (BFT), aquaculture systems that are near-zero water exchange systems, and that integrate effective biomass within the production volume. Integrated biomass in aquaculture production includes converting both waste feed and waste excreted from culture species to a resource with a positive influence on health and production performance parameters, resulting in increased economic value (Paerl & Pinckney 1996; Azim & Little 2006; Crab *et al*. 2012; Martínez-Córdova *et al*. 2015; Nevejan *et al*. 2018; Khanjani & Sharifinia 2020; Biswas *et al*. 2022; Khanjani *et al*. 2023).

High specific surface area media with high void space can be used in IPT aquaculture, with an adjusted C:N ratio, to support the culture of heterotrophs in periphyton at the media surface. Consortia of autotrophs and chemotrophs reside in lower oxygen and flow conditions within the media with nonbacterial single and multicellular organisms to form part of the ecosystem on and within the media. The ecosystem optimally utilises waste feed and excretions for biomass production which then becomes available as live feed to omnivorous species (Martínez-Córdova *et al*. 2015; Biswas *et al*. 2022). Carbon addition supports fast-growing heterotrophic organisms, increasing the capacity for the management of water quality, health improvement and waste recycling (Martínez-Córdova *et al*. 2015, Hasan *et al*. 2012; Asaduzzaman *et al*. 2010).

A Recirculating Aquaculture System (RAS) typically includes a solid removal clarifier and a biofilter that is external to the aquaculture production volume and requires flow to be pumped through the external system (Bell *et al*. 2023; Taufik *et al*. 2024), while also being a low water exchange system. Mechanical energy consumption, additional complexity outside of the production volume and additional infrastructure area of RAS are costly. IPT displaces the external biofilter required by RAS and the need to circulate water through a biofilter, while biomass waste discharge is reduced or eliminated (Crab *et al*. 2007). IPT also maintains the bulk of biomass on the media surface as periphyton rather than as biofloc, which must be kept suspended and requires solid management, as in BFT. The advantages of IPT with respect to complexity, cost, and compactness have been addressed in prior studies (Schryver *et al*. 2008). Among these three systems, IPT is an innovative zero-discharge system that generates the least amount of residual solids from equivalent production, without footprint or mechanical equipment external to the production volume.

The positive impact of IPT biomass includes an improved survival rate (SR) and product quality. Pathogen deterrence occurs through promotion of beneficial bacterial populations, inducing both quorum sensing and probiotic action, with periphyton as a source of live feed, enzymes and other beneficial compounds (Crab *et al*. 2007; Khanjani *et al*. 2022). In addition, the use of IPT media within the production volume increases the domain area as the media surface becomes available to prawns for grazing and for safer ecdysis (Coyle *et al*. 2010; Tuly *et al*. 2014). The use of IPT media is designed to achieve simultaneous water quality management, domain improvement and nutritional and health benefits. Biswas *et al*. (2022) demonstrated a reduction in supplementary feeding with improved sustainability in brackish water polycultures utilising added substrate to increase the quantity of available periphyton. Kumar *et al*. (2019) and Audelo-Naranjo *et al*. (2010; 2011; 2012a; 2012b) used Aquamat™, a substrate with characteristics like the Matala™ mat used in the present study, to rear whiteleg shrimp, *L. vannamei*, in a periphyton aided system in saltwater. IPT has been researched in freshwater applications but less than in saltwater and brackish water applications in comparison with BFT and RAS. Despite the traceable path of development of IPT, there is a gap and there remains a lack of consistency in application, i.e., in treatment of specific area of media, between research studies.

The giant freshwater prawn *Macrobrachium rosenbergii* is an omnivorous, detritivorous, and caprophagous freshwater species that grazes readily on periphyton organisms (Marlowe 2006). As such, *M. rosenbergii* culture is well suited to study of IPT. M. rosenbergii is readily marketable at sizes above 35 grams per prawn, but commercial aquaculture is most effective when a uniform product is produced. Uniform product output from cultures of mixed sex *M. rosenbergii* is impacted by the sex ratio, where the growth of females is distinct from that of males, and also by heterogeneous independent growth (HIG) of males. Karplus (2005) described the social hierarchy that inhibits the growth of the bulk of the culture population. A group of males, blue claw (BC) males, develop large claws, whereas lower-ranking orange claw (OC) males have a smaller claw size and small males (SM) have an even smaller claw size. Clawed *M. rosenbergii* exhibit cannibalism (Karplus 2005). The growth rate of males decreases from BC to OC to SM throughout a full typical cycle duration, during which females reach a maximum of about 35 grams (Karplus 2005). In the nursery stage, up to 45 and even 60 days, preceding claw development, *M. rosenbergii* are relatively homogenous in size and growth compared with the subsequent growth period. The literature lacks studies distinguishing nursery and grow-out phases for continuous culture of the species. Daniels and D’Abramo (1994) established that size separation at 30% of larger and 70% of smaller prawns led to significantly greater mean body weights (BWs) and average yields at 125–138 days.

Haslawati *et al*. (2022) reported that *M. rosenbergii* farming in Malaysia had a low impact on the environment, which was an assessment of the status quo of extensive farming, with its low output per hectare. To improve the national output of only 350 mt year^−1^ in 2018 (Hamzah *et al*. 2022) and productivity ranging from 0.9 to 3.15 mt ha^−1^ cycle^−1^ (Habib 2017; Banu & Christianus 2016), intensive farming of *M. rosenbergii* needs to match the sustainability of low-output extensive farming. IPT is a zero-discharge aquaculture system that holds promise for the sustainable aquaculture of *M. rosenbergii* at relatively high levels of productivity on existing or expanded farm bases.

This study aimed to evaluate the viability and effectiveness of Integrated Periphyton Technology (IPT) as a sustainable aquaculture system for culturing mixed sex *M. rosenbergii*. Specifically, we sought to:

1. Compare the growth performance, survival rates and feed efficiency of prawns reared in IPT versus a recirculating aquaculture system (RAS) during a 44-day nursery phase.
2. Assess the potential of IPT for commercial-scale application through a subsequent 60-day grow-out phase.

This study demonstrates the acceptability of IPT as a resource-efficient alternative to extensive aquaculture. It is significant in delivery of direct comparison of RAS with IPT. Use of periphyton biomass cultivated on Matala™ substrate within the production volume is a novel technique with potential for implementation in a wide range of tanks and ponds, where it can enhance production outcomes while minimising environmental impact.

## MATERIALS AND METHODS

The experimental trials of M. rosenbergii in IPT began with a density of 1,000 m^−2^ for trial T1, 44 days of nursery culture with extraction of a sample set, with a total of 104 days of culture. Three-dimensional media was supported within each production volume for IPT. The same density was applied in T1 in RAS. An equivalent quantity of media to IPT production volume media was provided within each RAS biofilter tank for the triplicate RAS control.

An additional 60 days of culture in trial T2 extended the evaluation to post nursery. Nursery harvest populations were subjected to a selection process for prawns below an approximate Body Weight (BW) of 1 g as the selected population of lower density for culture in the T2 grow-out stage. The method of separation employed was a visual assessment of size combined with batch weighing. This method of separation led to different densities between tanks for the T2 grow-out stage. In T2, individual tanks of IPT and RAS contained a set of size-selected, smaller prawns for continued culture in the original tanks (employed in T1) for an additional 60 days of growth. Performance data, including Feed Conversion Ratio (FCR), Body Weight Gain (BWG) of individuals and the total population, and the Survival Rate (SR) were recorded to evaluate IPT performance in comparison with RAS in the nursery phase. The substrate arrangement for IPT and RAS was unchanged from T1 to T2, and the specific area of periphyton media in the IPT production tanks was equivalent to that provided in the RAS biofilter tanks. SR, BWG, Average Daily Gain (ADG). FCR achieved versus applied supplementary feed were observed at harvest after 104 total days of culture. BWG, population counts, ADG, and SR were used to evaluate IPT performance relative to RAS for T2.

The removed set of larger prawns, those above an approximate BW of 1 g, were cultured as a composite group in a single IPT tank as T3 for a growth duration of 60 days, in parallel with T2. The relative performance at T2 and T3 was also evaluated. A comparison of the harvested populations of male morphotypic varieties and females was also possible at T2 and T3, with fast visual identification at 104 days of total culture, with respect to BC, OC and SM male and female individuals (Ra’anan *et al*. 1991; Karplus 2005).

### Experimental Setup

**FIGURE 1:**
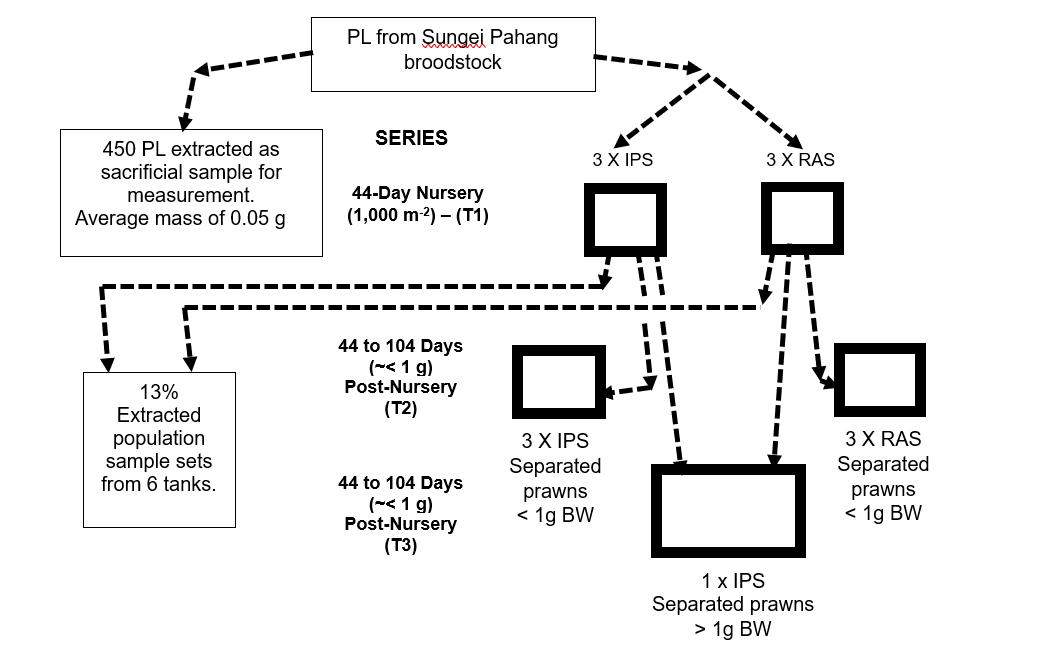
Experimental organisation of the 44-day nursery and 60-day post nursery culture in RAS and IPT tanks.

Fig. 1 shows the experimental organisation of the 44-day nursery (T1) and 60-day post nursery (T2 and T3) cultures of *M. rosenbergii*. The nursery PL for the experiment originated from the AKUATROP hatchery utilising broodstock obtained from Sungai Pahang, Pahang, Malaysia, from which a separated, sacrificial sample of 450 pieces of hatchery PL was measured. For T1, the three tanks in IPT (IPT1, IPT2 and IPT3) and RAS control (RAS1, RAS2 and RAS3) each received 1,500 pieces of hatchery PL, which amounted to densities of 1,154 individuals m^−3^ and 1,000 m^−2^ of tank footprint, in each tank. Water quality measurements throughout operation included daily dissolved oxygen, temperature, TDS and pH measurements via a handheld probe [Yellow Springs Instrument - YSI Professional Plus].

**FIGURE 2:**
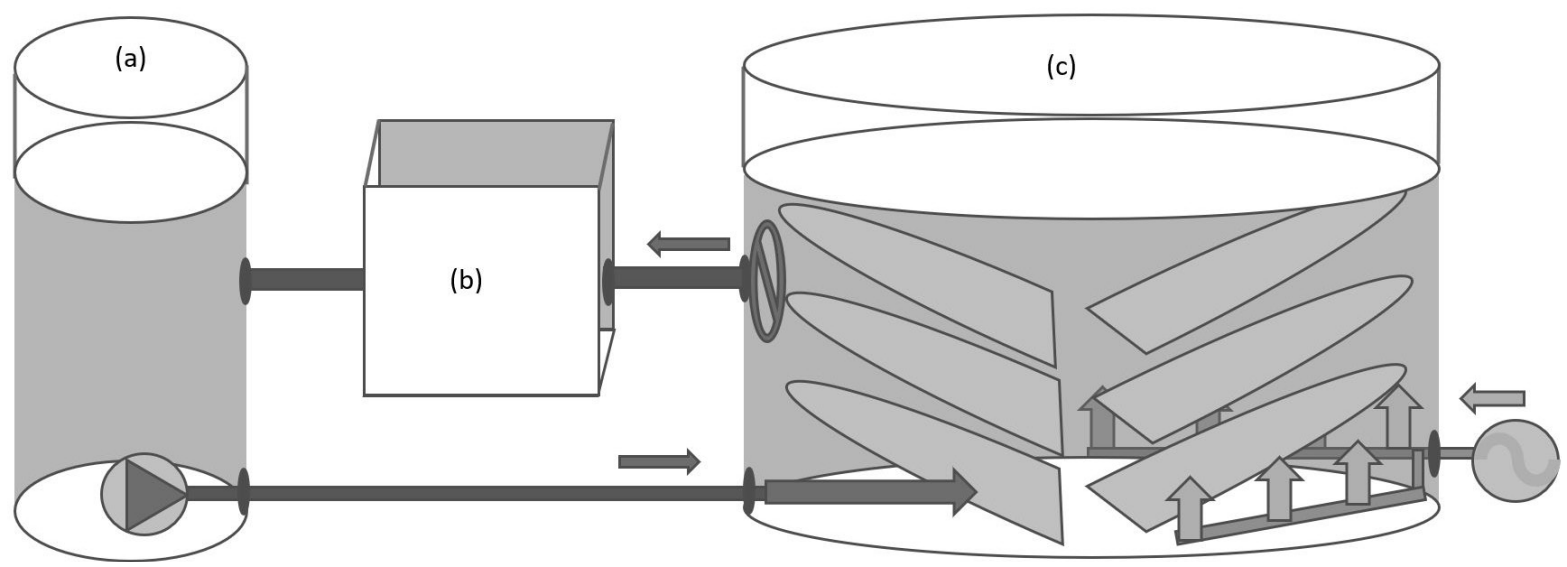
Diagrammatic layout for the treatment and control groups: (a) pump tank to RAS tanks only, (b) biofilter for RAS tanks only, (c) production volumes containing high specific surface area fibre mats for IPT and 1 cm HDPE open mesh for RAS, each totaling 3 m2 single-sided area.

The T1 treatment was operated under zero water exchange with small replacement for water evaporation, as were the T2 and T3 (designated IPT_Large_) treatments. T1 and T2 tanks of 1.3 m^3^ effective volume and 1.5 m^2^ bottom area were employed (diameter of 1.4 m and effective depth of 0.85 m) for IPT and RAS production volumes in T1 and T2. A diagrammatic arrangement of the IPT and RAS systems of T1 and T2 is depicted in Fig. 2. The IPT arrangement at T3 was also consistent with that shown in Fig. 2. The three RAS tank biofilter elements, T1 and T2, labelled (b) were established in fibreglass tanks of 1.2 m × 1.2 m × 0.8 m with 0.6 m effective water depth, in which water flowed through three sequential, permeable walls of Matala™ media. Each IPT and RAS production tank and each RAS biofilter tank were aerated with ceramic diffusers supplied from a common compressor at a maximum of 60 L min^−1^ per tank. A substrate with a high specific surface area (300 m^2^/m^3^) and high void ratio (94%) was suspended such that 200% of the tank bottom area (single side) of the media external surface area was provided within the T1 and T2 IPT production volumes. The RAS external biofilters contained identical media quantities and specific and external surface areas as those employed in IPT to maintain the water quality in RAS. The RAS production volume tanks all utilised open 1 cm mesh screen layers at 200% of the culture tank bottom area (single side) with a low specific surface area. Each RAS system was provided with a submersible pump of 6 m^3^ h^−1^ capacity in a biofilter overflow pump tank, producing circulation back to the RAS production volume.

The key characteristics of the IPT and RAS systems employed in the nursery and grow-out stages for T1 and T2, respectively, included 27 m^2^ of specific surface area provided per IPT tank and per RAS biofilter. In the IPT tanks, a submerged domain surface area of 6 m^2^ (two sides) was present in the production volume, whereas it was present only in the biofilter tank associated with each RAS tank. Additional domain surface area in the RAS production tanks was provided with mesh of low specific surface area. In T3 (IPT_Large_), 96 m^2^ of specific surface area and 16 m^2^ of domain surface area (two sides) were provided in the production volume via Matala™ media. The biofilter model applied to develop the substrate provisions followed Losordo and Hobbs (2000).

### Experimental Method

Fertilisation of T1 nursery tanks was carried out in advance of stocking with a dosage of 100 g of fertiliser (Serbajadi brand with N 12%, P 12%, K 17% and Mg 2%, and trace Mn, Fe, Zn, S, B, Cu and Mo) to each tank, along with 50 g of liquid organic fertiliser (Mr. Garrick), and the systems were aerated for eight days. There was no opportunity for refertilisation in the transition between the T1 nursery and T2 post nursery, so the tanks were refilled to 60% with the previous water from the zero discharge operation and topped up with dechlorinated water. The media substrate was not cleaned between the nursery and post nursery operations.

The feed schedule for the T1 nursery stage was developed from discussion with individuals experienced in M. rosenbergii aquaculture. For all tanks an average initial Post Larvae (PL) mass of 0.03 g was utilised, then providing 3% of the estimated surviving prawn mass as feed per day. An 80% SR was assumed to be distributed linearly over 44 days, which was the period selected for arrival of functional claws in the logistical schedule. A BW of 2 g was used for surviving prawns after 44 days of culture. Feeding was performed twice per day, at 9:00 and 17:00, using PL crumbles (Gold Coin Specialities) with a minimum crude protein content of 40%, maximum crude fibre content of 3%, minimum crude fat content of 7%, and no added antibiotics. Molasses was added as a carbohydrate source to maintain a C:N ratio of 20:1 (Browdy *et al*. 2012). Sodium bicarbonate (alkalinity) was added as 13% feed (Furtado *et al*. 2015), dissolved and delivered with dissolved molasses once per day.

For the T2 post nursery stage, an average BW of 0.62 g was calculated for the total transferred population, and the average number of prawns per tank, 581, was used as the starting point for the feed model. The SR assumed by the feed model for the 60 days of culture was 80%, and the 20% mortality was distributed linearly over the 60 days, with an average BWG of 2 g. The feed model provided 3% of the modelled, cultured prawn mass per day. The ratio of daily feed and carbohydrate addition to supplementary feed was determined at the nursery stage. The feed schedule developed for T3 IPS_large_ assumed a harvest BW of 7 g, utilised an average initial PL mass, provided 3% of the estimated surviving prawn mass per day, and assumed 80% SR linearly over the 60 days of culture.

The nursery stage harvest occurred at a total culture period of 44 days. Approximately 13% of the total population was extracted as sacrificial samples for measurement. Another approximately 7% of the total population was lost to attrition due to stress of separation and selection. These population losses were not considered to achieve a sufficient reduction in density for T2 for the set of 3 IPT tanks and 3 RAS tanks. The criterion used to reduce the population was the removal of large prawns, approximately above 1.0 g BW, from the T2 experimental population (Daniels & D’Abramo 1994). A group of prawns was weighed and measured to provide a basis for visual size appraisal of prawns above and below approximately 1 g BW. The small samples constituted 530, 782 and 696 individuals from IPS1, IPS2 and IPS3, respectively, and 829, 891 and 797 from RAS1, RAS2 and RAS3, respectively. The small prawn populations constituted the post nursery stocking of the IPS and RAS tanks and totalled 3,482 prawns.

Larger prawns harvested from T1, approximately above 1 g BW, constituted 397, 405 and 313 individuals from IPS1, IPS2 and IPS3, respectively, and 235, 293 and 304 from RAS1, RAS2 and RAS3, respectively, totalling 1,947 prawns. These prawns formed the composite group, which populated T3 (IPT_Large_), with a volume of 8 m^3^, at the beginning of 60 days of culture. At T3, attrition due to stress and sampling led to a final stocking population of 1,558, for a stocking density of 243 m^−3^. A total of 5,040 total stocked prawns were distributed to T2 and T3 at 69% and 31%, respectively, which was similar to the 30% size separation of larger prawns reported by Daniels and D’Abramo (1994).

Prawns were harvested from T2 and T3 at 104 days when HIG enabled the rapid visual distinction of male morphotypes and females. The harvested prawns were identified by male morphotype and sex and weighed individually, separately for each tank. The sex assessment was based on the identification of three male morphotypes, namely, small male (SM), orange clawed (OC) male and blue clawed (BC) male, with the remainder identified as female. The water quality parameters of temperature, dissolved oxygen (DO), total dissolved solids (TDS) and pH were measured with a portable handheld meter (YSI Professional Plus) for both the nursery and post nursery stages.

### Statistical Analysis

In both T1 and T2, the IPT and RAS treatments were distinguished only with respect to the location of periphyton on substrate media sufficient to maintain water quality. The RAS production tanks had domain areas equivalent to the media area available via IPT and were produced with low specific area open mesh to remove the domain area factor from the evaluation.

Statistical analysis was conducted on both the BWG of prawns harvested from each tank and the SRs experienced by individual tanks. The average BWG alone describes the mean mass of prawns produced, and BWG × SR measures the average production of individual tanks at harvest as the total mass in both the nursery and post nursery stages. The analysis was performed via Welch ANOVA and Games–Howell post hoc analysis via SPSS (Statistical Package for Social Science version 29.0). Significance was assigned at the 0.05% level. Two-way ANOVA was employed for analysis of the morphological and sex results together with the BWG and BWG × SR results.

The expression of HIG over 60 104 days of culture was then observed for the individual IPS and RAS tank populations of T2. Once morphology and sex could be assessed at harvest at 104 days of culture, HIG expression in SM, OC and BC males and sex became dependent variables in the statistical analysis for T2 and T3. The associations of BW and BWG with morphotype- and sex-dependent variables in post nursery prawns were assessed for comparisons of averages and significance of differences specific to the two treatments and experimental size groups.

## RESULTS

### Water Quality for T1 Nursery and T2 and T3 Growout Cultures

The water qualities measured for the IPT tanks and RAS tanks included temperature (°C), DO (mg/L), TDS (mg/L), and pH. These values were taken daily with a handheld meter (YSI). Results for temperature ranged from 27.5°C to 29.3°C, for DO from 6.3 mg/L to 8.3 mg/L, for TDS from 202 mg/L to 437 mg/L, and for pH from 7.2 to 7.6.

The total ammonia nitrogen (TAN) and nitrogen nitrite (N-NO_2_^−^) levels were monitored biweekly via API® test kits at T1, T2 and T3, which indicated that the levels were maintained below the minimal detection level of the test (0.025 ppm) throughout the 104-day trial period.

### Performance Indicators

#### Performance indicators for T1 nursery culture harvest

The total feed delivered was used in the determination of FCR. The calculated SR, BW, harvest density (kg m^−3^ and no. m^−2^), BWG, ADG and FCR, including standard deviation (SD), are reported as averages for all the tanks in each treatment set in Table 1. SRs were generated from complete counts, but the BW data reported were determined from a subsample composed of approximately 13% of the T1 harvested prawns.

#### Performance indicators for T2 and T3 post nursery growth

The total feed provided was used in the determination of FCR. The calculated SRs, BWGs, ADGs and FCRs are reported as averages, with gender data, for all the tanks in each group in Table 2.

### Statistical Analysis of T1, T2 and T3

The data are presented as the means ± standard deviations unless otherwise stated.

#### Statistical analysis for T1 nursery culture

A total of 450 postlarvae (PLs) were measured. Violation of normality was found for each sample, and 8% of the outliers in both datasets were assessed via boxplot inspection. With outliers removed, the sample mean BW was not significantly different from the normal mean of 0.0287, *t* (413) = −2.512, *p* = 0.012, Hedges’ g = 0.123 (95%, −0.22, −0.027).

Examining the T1 harvest data to determine if BW and BW × SR were significantly different for the IPT (treatment) and RAS (control) groups via SPSS version 29, boxplot assessment found 10.5% outliers, and the assumption of a normal distribution was violated for each treatment group. The homogeneity of variances was violated, as assessed by Levene’s test of homogeneity of variance (*p* < 0.001). The large sample “n” was a representative random sample consisting of approximately 13% of the full population at nursery harvest. BW and BW × SR at harvest of the sample were evaluated for significant difference between the IPT and RAS groups (*p* < 0.05). Table 3 summarises the BW and BW × SR means with standard deviations for the T1 harvested samples. The mean T1 BW at harvest increased from RAS2 (*n* = 118, 0.70 ± 0.48) to IPT2 (*n* = 119, 0.71 ± 0.50), IPT3 (*n* = 101, 0.87 ± 0.67) to RAS1 (*n* = 107, 0.93 ± 0.74), RAS3 (*n* = 68, 1.13 ± 0.95) and IPT1 (*n* = 93, 1.24 ± 0.77). The BW at harvest was significantly different for IPT and RAS, Welch’s F (5, 256.118) = 10.137, *p* < .001. Table 3 also summarises the results of the analysis for BW in IPT and RAS for T1 nursery culture. Within groups, the highest performing tank pairs of IPT tanks and of RAS tanks, the lowest performing IPT tanks and RAS tanks, and the mid-range performance IPT tanks and RAS tanks were not significantly different across pairs.

The mean BW × SR at the T1 harvest increased from RAS2 (*n* = 118, 0.55 ± 0.38) to IPT2 (*n* = 119, 0.56 ± 0.40), IPT3 (*n* = 101, 0.59 ± 0.45) to RAS1 (*n* = 107, 0.66 ± 0.53), IPT1 (*n* = 93, 0.77 ± 0.48), and RAS3 (*n* = 68, 0.83 ± 0.70). The BW at harvest was significantly different for IPT and RAS, Welch’s F (5, 258.733) = 4.495, *p* < .001. The group means were significantly different (*p* < .05) between the complete IPT and RAS groups. The IPT tanks performed equivalently to the RAS tanks, pairwise, with IPT2 results close to RAS2, IPT3 results close to RAS1, and IPT1 results close to RAS3, although RAS3 BW × SR was significantly greater than that for IPT1. In the nursery study, IPT was considered able to establish equivalent performance in comparison with RAS.

**FIGURE 3:**
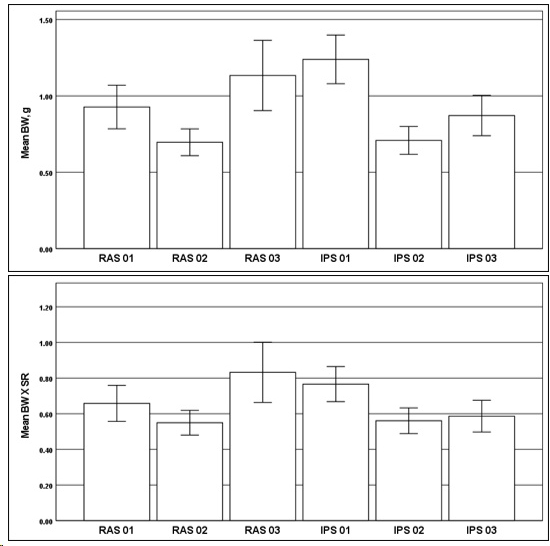
Comparison of means of BW (top) and BW × SR (bottom) for T1 nursery culture individual prawn measurements with ranges for the tank sample populations.

A comparison of the means of BW and BW × SR of harvested prawns from T1 nursery culture is shown in the charts presented in Fig. 3 (top) and (bottom). These charts present the range of individual prawn measurements with ranges for the sample population. The charts provide valuable insight into the variation experienced in the nursery stage, which has implications for grow out.

#### Statistical analysis for T2 post nursery culture

After separation of a representative sample of approximately 13% of the T1 harvest, the harvest was separated visually to remove prawns > 1 g from the T2 stock. After size separation, the prawns designated for T2 stocking were sampled and weighed as IPS1, IPS2, IPS3, RAS1, RAS2 and RAS3 samples of 53, 78, 71, 83, 89 and 80 individuals, respectively. Analysis was performed to determine if BW was significantly different for the IPS (treatment) and RAS (control) groups of the starting populations of each tank (*p* < 0.05) which found the assumption of a normal distribution was violated for both groups. Homogeneity of variances was violated.

Further analysis determined if the calculated BWG and calculated BWG × SR were significantly different for the IPS (treatment) and RAS (control) groups, and to further evaluate differences between tanks in each group. The outliers, as assessed by a boxplot, were 1.3% for BWG and 1% for BWG × SR. Homogeneity of variances was violated for both BWG and BWG × SR.

**FIGURE 4:**
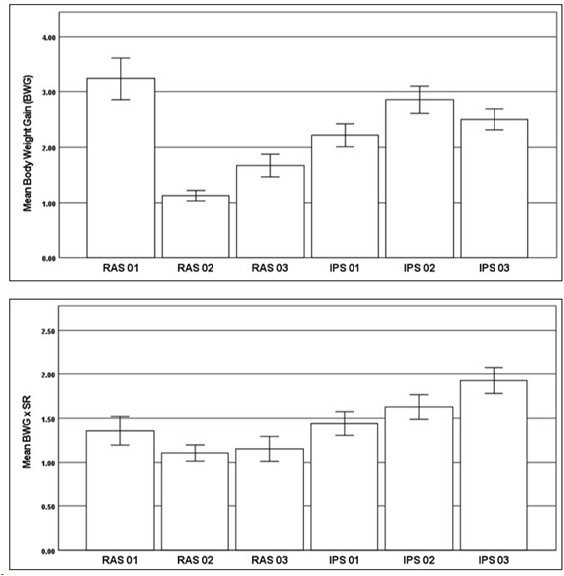
Comparison of means of BWG (top) and BWG × SR (bottom) for T2 individual prawn measurements with ranges for the tank sample populations. IPT was significantly different, with higher means of BWG × SR than RAS.

The mean BWG at harvest increased from RAS1 (*n* = 259, 3.24 ± 3.13) to IPS2 (*n* = 342, 2.85 ± 2.29), RAS3 (*n* = 445, 1.67 ± 2.19), IPS3 (*n* = 402, 2.50 ± 1.93), IPS1 (*n* = 258, 2.22 ± 1.67), and RAS2 (*n* = 691, 1.13 ± 1.25). BWG did not appear to be correlated with “n”, except negatively for RAS. The BWG group means were significantly different (*p* < .001). The IPS tanks did not consistently display increased BWG figures over the RAS tanks as a set within the group. BWG × SR at harvest increased from RAS2 (*n* = 691, 1.11 ± 1.22) to RAS3 (*n* = 445, 1.15 ± 1.51), RAS1 (*n* = 259, 1.36 ± 1.32), IPS1 (*n* = 258, 1.44 ± 1.09), IPS2 (*n* = 342, 1.63 ± 1.30), and IPS3 (*n* = 402, 1.93 ± 1.48). The BWG × SR ratio at harvest significantly differed between the IPS treatment group and the RAS control group. A comparison of the means of BWG and BWG × SR of prawns harvested from individuals, postharvest IPS tanks and RAS tanks is shown in the charts presented in Fig. 4. Compared with RAS, IPS improved performance with respect to BWG × SR. These charts present the range of individual prawn measurements with ranges for the sample population. The charts provide valuable insight into the variation experienced in grow out.

### Morphological and Gender Evaluation of T2 and T3 Post Nursery Culture

The total feed provided was used in the determination of FCR. The calculated SRs, BWGs, ADGs and FCRs are reported as averages, with gender data, were provided for T2 and T3 in Table 2.

The feed model for T2 resulted in a total of 1,530 g of supplementary feed being applied over 60 days, and the group average FCRs realised in the IPT and RAS tanks were 1.79 and 1.88, respectively. For the single IPT_large_ tank of T3, the feed model supplied 11,113 g of supplementary feed, and the FCR was 2.16 over 60 days to harvest.

**FIGURE 5:**
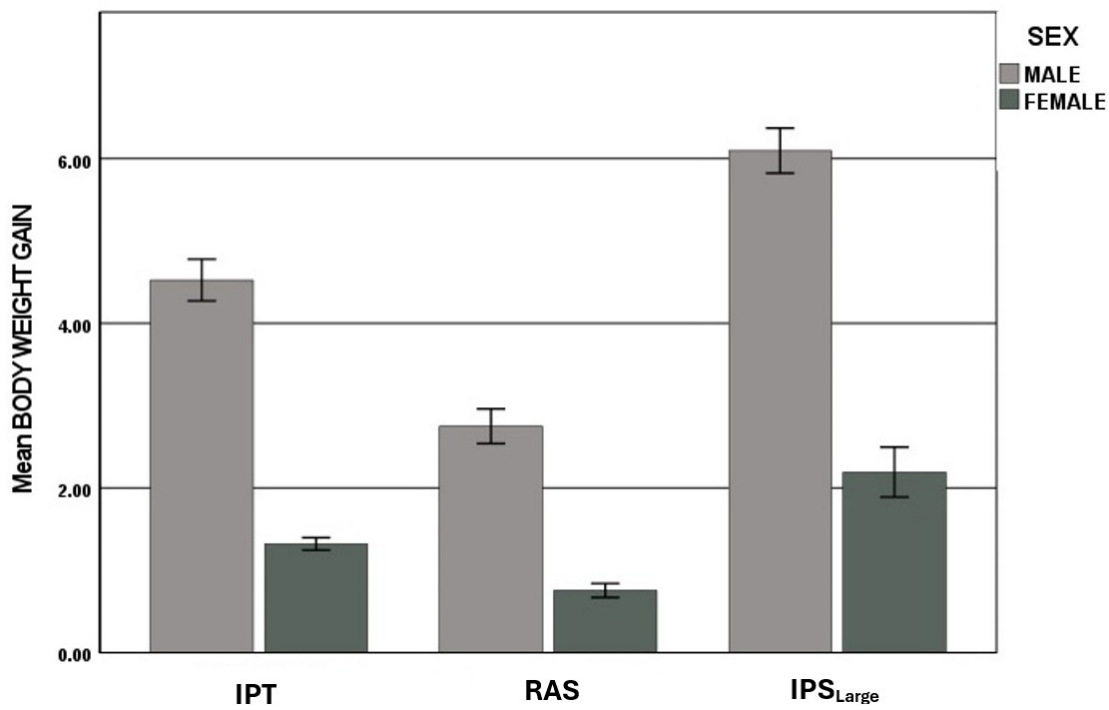
Comparison of means of BWG for T2 and T3.

The BWGs of harvested prawns from T2 IPT and RAS and T3 IPT_large_ are given in Fig. 5, reflecting the standard deviation of the total population. The difference in the mean BWG between males and females was significantly greater at harvest for IPT than for RAS. For both T2 and T3, the BWG of males was significantly greater than that of females. The subset stocked to IPS_large_ demonstrated that the larger prawn subset maintained a growth advantage through the additional 60 days of culture for both male and female prawns.

**FIGURE 6:**
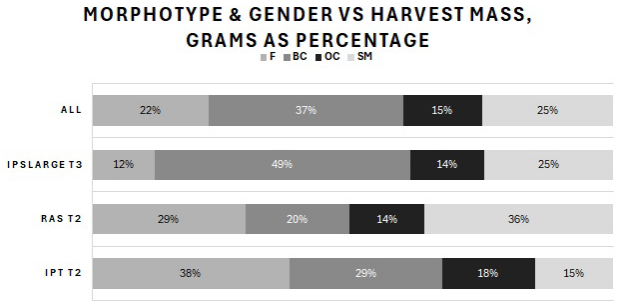
Comparison of average percentages of total harvest BW for three male morphotypes and females displayed in the identical order as in the legend.

**FIGURE 7:**
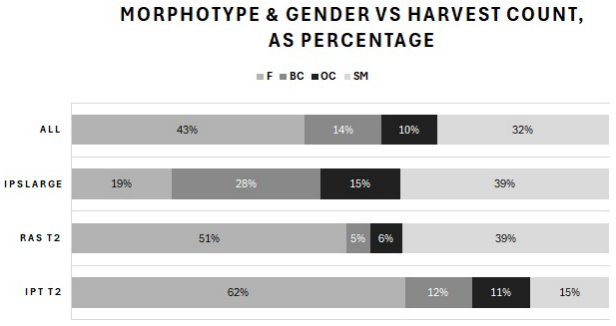
Comparison of average percentages of total harvest population counts for three male morphotypes and females displayed in the identical order as in the legend.

**FIGURE 8:**
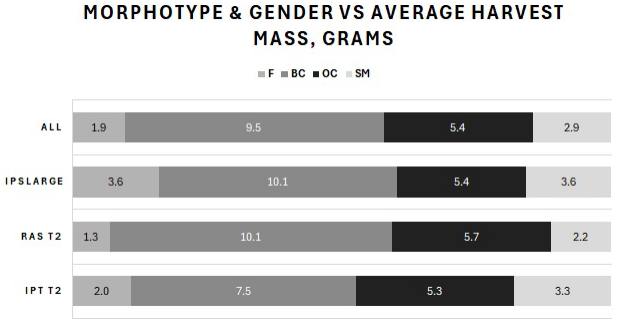
Comparison of average BW among the three male morphotypes and females displayed in the identical order as in the legend.

**FIGURE 9:**
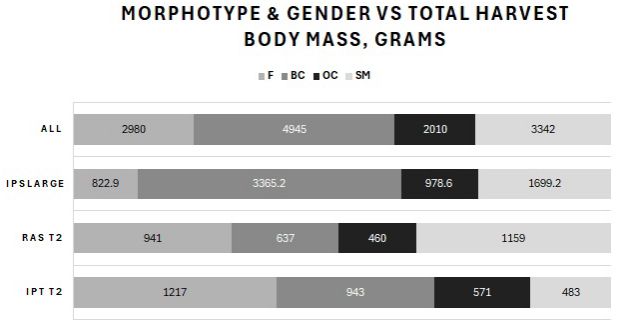
Comparison of average BW among the three male morphotypes and females displayed in the identical order as in the legend.

Harvested prawns from the T2 (small, < 1 g BW) and T3 (large, > 1 g BW) outputs were classified by sex and three male morphotypes (SM, OC and BC males) as percentages by BW and population count, average BW and total harvest mass per tank. The results for the summed total population of all tanks (ALL) included the harvested prawns from both T2 and T3. The percentages by BW are shown in Fig. 6, and the number of surviving prawns is shown in Fig. 7. Fig. 8 shows the average BW in grams of surviving prawns, and Fig. 9 shows the total harvest biomass in grams of surviving prawns. These prawn populations represent the output at 104 days of culture of prawns harvested from the T1 nursery trial, minus the total of approximately 20% attrition incurred for sampling and mortality during post nursery transfer.

The percentages of BW at harvest for each male morphotype and females of the total BW produced in each treatment, and for the overall population are presented in Fig. 7. The final BW achieved as BC at T2 IPT was 29% versus 20% for RAS. BC inclusive of OC for T2 IPT produced 47% of total harvest mass compared with 34% for T2 RAS. T3 produced 49% as BC and a further 14% as OC. Harvest mass of females plus SM was 43%, 65%, 37% and 47% for IPT, RAS, T3 and the total population (ALL), respectively.

The count of individuals at harvest for each male morphotype and females of the total count produced in each treatment, and for the overall population are presented in Fig. 8. The final count achieved as BC at T2 IPT was 12% versus 5% for RAS. BC inclusive of OC for T2 IPT produced 33% of total harvest count compared with 11% for T2 RAS. T3 produced 28% as BC and a further 15% as OC. Harvest count of females plus SM was 77%, 90%, 38% and 75% for IPT, RAS, T3 and the total population (ALL), respectively.

The total harvest body mass of individuals at harvest for each male morphotype and females produced in each treatment, and for the overall population are presented in Fig. 9. The total harvest body mass achieved as BC at T2 IPT was 943 g versus 637 g for RAS. BC inclusive of OC for T2 IPT produced 1,514 g of total harvest body mass compared with 1,097 g for T2 RAS. T3 produced 3,365 g as BC and a further 979 g as OC. Total harvest body mass of females plus SM was 1,700 g (27% of total harvest body mass), 2,100 g (33% of total harvest body mass), and 2,522 g (40% of total), for IPT, RAS, T3 and the total population (ALL), respectively. Total harvest body mass of the population was 6,322 g.

Table 4 displays the Specific Growth Rate (SGR) values calculated for each tank over the culture duration and for the total T2 and T3 populations (ALLs).

**FIGURE 10:**
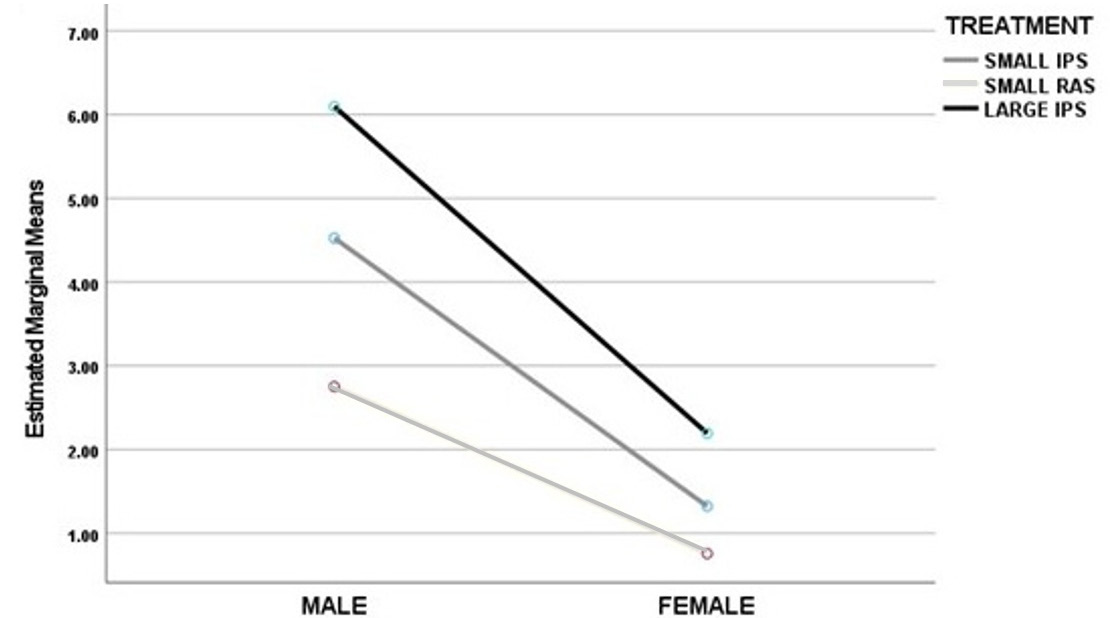
Comparison of male and female BWG in grams by treatment group.

At 104 days total DoC, females and the three male morphotypes from size-separated groups were harvested and visually identified. Harvested prawns were weighed and assessed for male morphological features distinguishing between BC, OC and SM and between sexes. Analysis was conducted to examine the effects of sex and treatment type (IPS and RAS of small stocks in triplicate in T2 and IPS_large_ at T3) on BWG that showed a statistically significant difference in the mean BWG scores between males and females in the treatment groups. Fig. 10 displays the post nursery trends of BW for males and females at harvest, averaged for each treatment of smaller and larger prawns.

## DISCUSSION

IPT provides water quality control and exposes cultured animals to periphyton as a nutritional and health aid. IPT does so without the external cycling and treatment processes required for RAS. Zero discharge in T1 and T2, for both IPT and RAS, did not result in an increase in TDS above 500 mg/L over the 44-day nursery and 60-day growth trials, and issues with water quality were not observed. A number of studies utilising c media have demonstrated that nitrogen species are typically maintained within boundaries of adequate conditions for the farming of aquaculture species, including both marine (Audelo-Naranjo *et al*. 2010; 2011; 2012a; 2012b; Huang *et al*. 2013) and freshwater species (Arndt *et al*. 2002; Yu *et al*. 2016; Li *et al*. 2019).

*M. rosenbergii* exhibits heterogeneous individual growth (HIG) among males, with females also differentiated in their growth curves, and these influences impact commercial production of uniform products. Heterogenous individual growth (HIG) has been identified as having a major impact on successful aquaculture of the species, mainly due to the lack of uniformity required for effective marketing of production (Ranjeet & Kurup 2002). The HIG does not display until the presence of claws in males; hence, it is not considered as important in nursery culture as it is in growing out. BC male partial harvesting typically begins after 60 days (Rahman *et al*. 2010), which is also when higher-growth individuals referred to as “jumpers” express relatively high relative growth rates (Karplus 2005), and prior to 45 days, the impact of BC males on the survival and growth of other morphotypes is less significant. Daniels and D’Abramo (1994) and Daniels *et al*. (1995) separated the nursery *M. rosenbergii* by size, selecting a grid mesh ahead of grow out. Post-nursing size selection did not eliminate heterogeneity in the separation of recently metamorphosed PLs but did have an impact after 50 days of nursery culture at harvest. The size separation that appears within the nursery phase can be utilised to increase the accuracy, with improved product uniformity (Coyle *et al*. 2010). Size separation of nursery prawns was applied ahead of the grow out trial, which resulted in counts of prawns of 69% for the smaller prawns and 31% for the larger size-separated prawns. During the transition from nursery T1 to post nursery T2, size separation coincidentally established a lower density for the post nursery phase. The technique of size separation was applied to the output from triplicate IPT and RAS tanks, and the hierarchy of growth that had developed in the nursery phase was preserved in T2 post-nursing, with 69% of the prawn population. With the different densities applied to each tank in T2, the RAS tank densities were generally higher than those for the IPT tanks at the beginning of T2. Compensatory growth is considered to effectively balance experimental effects at T2 (Marques *et al*. 2010; 2012; Marques & Lombardi 2011).

Nursery culture is receiving increased attention for multiple species under aquaculture because of its ability to control the initial trial of growth within highly controlled conditions at higher densities than those employed for growth. Coyle *et al*. (2010) reported a convincing case in which the implementation of a nursery stage increased resistance to predation, cannibalism and environmental conditions; improved the survival rate; and generally resulted in increased individual weight, production and harvest value. The ADG calculated for surviving prawns at harvest in T1 was 0.027 g d^−1^ for the IPT tanks and 0.026 g d^−1^ for the RAS tanks. The average survival rates for trial T1 were 69% for the IPT tanks and 74 g% for the RAS tanks. The BW achieved for trial T1 was 0.92 g for the IPT tanks and 0.88 g for the RAS tanks. The BW × SR for the nursery trial T1 were 0.063 g d^−1^ for the IPT tanks and 0.065 g d^−1^ for the RAS tanks, which were not significantly different. The final BW displayed pairwise equivalence between the triplicate IPT and RAS cultures in the T1 nursery stage. The FCR achieved in T1 was 2.02 for IPT and 1.97 for RAS in response to an FCR model feeding strategy of 1.04. T1 IPT and RAS were similar in terms of SR and BW and achieved FCR. Valenti and Moraes-Riodades (2004) reported an FCR of 2.50 for conventional clear water nursery systems. Ballester *et al*. (2017) reported FCRs over 30 days of culture of *M. rosenbergii* of 1.82 and 2.25 and SRs of 86% and 77% for RAS and BFT, respectively, with low stocking densities of 150 m^−2^. The lack of clear differentiation of results between IPT and RAS could indicate that IPT-integrated media biomass does not improve productivity in nursery culture on the basis of these criteria. The health and nutritional quality of the produced prawns were not examined. The SGR values for TI were 1.12% d^−1^ and 1.13% d^−1^ for IPT and RAS, respectively. Ballester *et al*. (2017) reported SGRs of 1.39% d^−1^ for RAS and 1.21% d^−1^ for BFT trials over 30 days of culture. The results for SR, SGR and FCR for the higher density applied at T1 (1,500 m^−2^) indicated the effective performance of both the IPT and RAS nursery systems.

The average harvest density for T2, after 60 days of growth, was 339 and 436 individuals m^−2^ for IPT and RAS, respectively. *M. rosenbergii* is typically cultivated at lower densities than marine prawns, both in nurseries and growing out. The highest growth densities of *M. rosenbergii* worldwide have been reported in Chinese aquaculture, with densities of up to 65 m^−2^, and consistent Malaysian densities of 15 m^−2^ have been reported (New & Kutty 2010), whereas the growth of *L. vannamei* has varied between approximately 250 and 500 m^−2^ (Ray *et al*. 2011; Suantika *et al*. 2018; Schveitzer *et al*. 2012; Zhang, Lin, Huang *et al*. 2010; Zhang, Lin, Wang *et al*. 2010; Zhang 2011; Zhang *et al*. 2020). T2 IPT and RAS growth were conducted at densities within the range of those employed for *L. vannamei*, with SRs of 66% and 72% for IPS and RAS, respectively. SR was correlated with greater density for both IPT and RAS triplicate tanks. The average SR for the T3 IPS_large_ was 78%. The overall population of T1, T2 and T3, considering approximately 20% losses for sampling and transfer mortality, was estimated to be approximately 74%. Marques and Lombardi (2011) reported survival rates ranging from approximately 65%–70% in *M. rosenbergii* in net pen systems stocked at densities ranging from 50 m^−2^–200 m^−2^. Crab *et al*. (2010) applied BFT with different carbon sources and reported a 75% survival rate. Asaduzzaman *et al*. (2008) reported survival rates ranging from approximately 63 to 72% while evaluating increases in the C:N ratio with the application of periphyton substrates. Pérez-Fuentes *et al*. (2013) reported survival values of 85% for rearing *M. rosenbergii* in both BFTs and traditional ponds. The SRs found in the current study are broadly similar to those reported in prior studies and were selected to represent the higher range of SRs previously achieved in comparable systems. An increase in the SR from post nursery size separation was not clearly demonstrated.

BWG × SR was examined by Welch ANOVA with Games–Howell post hoc tests to evaluate productivity. Significance was assigned at the 0.05% level. The results were examined for comparisons of the averages and significance of differences in BWG × SR for triplicates of each of the two groups, IPT and RAS, to evaluate the outcome on the hypothesis that IPT yielded at least equivalent productivity in comparison with RAS in the grow-out culture of *M. rosenbergii*. The group results for BWG × SR were significantly different. Triplicate IPT tanks were ranked as the top three with respect to BWG × SR, with triplicate RAS tanks ranked as the bottom three. Compared with RAS, IPT improved productivity.

The specific growth rate (SGR) for triplicate tanks with smaller prawns in T2, IPT_large_ with larger prawns in T3, and the overall population (ALL) was observed. IPT_large_ was populated by a subset of the largest nursery prawns and yielded a marginally higher SGR (1.91% d^−1^) than did the overall population (1.89% d^−1^), which was not considered significant. However, two out of three IPT tanks and one RAS tank of T2 each presented higher SGR values than did the total harvest population (ALL), whereas IPT_large_ was not significantly greater. Asaduzzaman *et al*. (2008) reported that the SGR for various C:N ratios and applications of periphyton substrates ranged from 1.56 to 1.75% d^−1^ after 120 days of culture. The SGR achieved by IPT and RAS via Matala™ media was greater than that reported in these prior studies. The improved SGR result was consistent for the range of nursery prawn subsets generated in the experiment. This result could support the selection of nursery prawns by size to produce a higher SGR, but the result could also be attributed to the adoption of Matala™ media supporting IPT.

The percentage distributions of the harvest count of females, SM, OC males and BC males over the whole harvest (ALL) were 43%, 32%, 10% and 14%, respectively. Karplus (2005) and Karplus and Sagi (2010) extensively reviewed evaluations of the proportions of male morphotypes and females in experimental populations. These proportions vary significantly with density. At low densities, the percentage of BC males has been reported to reach 20%, with that of small males decreasing to 33%. BC males are the desired market product but decrease in proportion at higher densities. The 40% group of larger prawns at T3, IPT_large_, presented a BC count of 28%, with a concurrent female prawn count of 19%. The group of smaller prawns in T2 ranged from 4 to 18% (IPT) and 2 to 7% (RAS) in triplicate. The results indicated that sex and morphology are likely related in terms of ratios before T1 44 days of culture. By extracting larger prawns, BC males were disproportionately transferred to T3. Over 60 days of culture, T2 prawns did not demonstrate morphological advancement in males from SM and OC males into BC males. Gender, morphological, and performance differences were used to evaluate the potential of the separation technique for commercial application. Further trials are needed to understand the economic benefits obtained through size separation.

The average harvest BW at 104 days of culture attributed to females and to SM, OC and BC males followed a similar hierarchy for T2 and T3 and for the total of all tanks (ALL). The ranges in BW for SM, OC and BC males were approximately 1.4 to 5.5 g, 4 to 7.5 g and 7 to above 12 g, respectively. The weight of the female prawns ranged from approximately 1.0 g to 2.0 g in all tanks, except for a maximum of 3.5 g in T3 IPT_large_. At 104 DoC, females performed no better than SM did. This result supports the use of all-male stock since the female population is lagging in growth but is consuming feed and may play a role in stimulating BC male conflicts (Karplus 2005).

The total prawn biomass at harvest, after allowing for losses in transition, designated ALL, was 13,434 g. There were approximately 5 kg of BC males, and almost 3.5 kg of those BC males (70%) arose in Phase B4 IPS_large_, which also had a total harvest mass of almost 7 kg. The average BW of BC males in IPS_large_ was approximately 10 g, which was exceeded by tank RAS1 of T2, for which BC males averaged above 12 g. The BW of BC males could not be said to be enhanced in any way by the separation of small and large nursery prawns.

It was acknowledged that statistical control was imperfect by some measures, yet statistical evaluation of the experimental events was a useful tool to investigate post nursery results against nursery stage results, to investigate the inherent relationships of the independent variable groups derived from a common batch of hatchery PLs and to evaluate the outcome on the hypothesis that IPT yields at least equivalent performance to RAS in the application of intensive *M. rosenbergii* culture. The observation of *M. rosenbergii* at the semicommercial scale is necessary, and the impact on the statistical analysis of the heterogeneous nature of the species, derived from both HIG and sex, must be considered.

## CONCLUSION

The comparative performance of IPT and RAS demonstrated that IPT displayed equivalent or better performance characteristics than RAS for 44 days for T1 nursery culture and for 104 days of total culture of *M. rosenbergii* and supported the IPT technological approach, which does not require an external effluent treatment system requirement and operates with zero water discharge. The periphyton biomass attached to the IPT media was able to maintain the water quality throughout the culture period, without the requirement for mixing energy for the suspension of biofloc as it operated as a clear water system.

The trial design distinguished the presence of periphyton within the IPT production volume from its use as an external biofilter in RAS. Providing additional surface area other than the pond bottom where *M. rosenbergii* typically resides provides prawn individuals with a safer location for ecdysis, which reduces hierarchical stress and the occurrence of cannibalism. However, the access of prawns in IPT to periphyton biomass was not conclusively responsible for the significant differences in BWG and BWG × SR between the IPT and RAS treatments. Future studies are needed to rationalise the use of IPT in postnursing culture of *M. rosenbergii*, including the evaluation of the quantities of media substrate required to maintain water quality, the minimum feasible FCR, the potential reduction in the impact of HIG, and the evaluation of the nutritional qualities of *M. rosenbergii* produced with IPT.

HIG makes the farm output of *M. rosenbergii* less profitable. Further investigations are needed to evaluate the uniformity derived from the partial harvesting of desirable BC males from size-separated populations of both mixed sex and all-male *M. rosenbergii* cultures. All-male culture is expected to produce significantly higher uniformity among male morphotypes, i.e., through the lack of female presence, reducing competition responses, but HIG is still active. The current study was unable to conclusively define how additional surface area might reduce HIG. Future research may evaluate mixed sex versus all-male cultures in IPT to determine whether significant improved performance and product uniformity can be achieved for culture of *M. rosenbergii*.

IPT deserves more definitive identification, and more attention for its further development. The application of IPT to ponds is important as a technique to rapidly convert extensive ponds to intensive ponds operating with nearly zero water exchange and a corresponding reduction in waste discharge. The general objective of this study was to prepare, operate, and characterise a representative IPT as a sustainable, intensive means of culturing *M. rosenbergii*. The findings of the present study increase the understanding of what can be accomplished with innovative application of IPT intensive aquaculture, and avenues of research can lead to economic improvement through more intensive production of *M. rosenbergii*.

## Figures and Tables

**TABLE 1:** Performance indicators for T1 nursery culture harvest.

Metric	Feed model	IPT, ave.	IPT (SD*)	RAS, ave.	RAS (SD*)
Harvest (N)	1,500	1,041	n/a	1,116	n/a
Survival rate (%)	100	69	9	74	4
Average BW (g)	1.29	0.92	±0.68	0.88	±0.73
BW x SR	1.29	0.63	±0.45	0.65	±0.53
ADG (g d^−1^)	0.028	0.020	n/a	0.019	n/a
Total feed per tank (g)	1,935	1,935	n/a	1,935	n/a
FCR	1.04	2.02	n/a	1.97	n/a

Note: SD* = Standard Deviation

**TABLE 2:** Performance indicators for T2 and T3, 60 days of post nursery growth.

Metric	IPT T2	RAS T2	IPT_Large_ T3
Input PL (N)	1,518	1,964	1,558
PL average BW (g)	0.67	0.58	1.46
Stocking density (g m^−3^)	262	288	290
Harvest (N)	334	465	1,214
Overall Survival Rate (SR) (%)	66	71	78
Males Harvested (N)	381	682	987
Females Harvested (N)	621	713	227
Ratio (Male:Female)	0.61	0.96	4.35
Harvest average BW (g)	3.3 ± 2.5	2.6 ± 3.1	5.7 ± 4.4
Male Harvest average BW (g)	5.6 ± 2.5	4.4 ± 2.3	6.1 ± 4.3
Female Harvest average BW (g)	1.9 ± 1.4	1.4 ± 2.0	3.6 ± 3.9
Harvest BWG (g)	2.6	2.0	4.24
ADG (g d^−1^)	0.04	0.03	0.07
Total feed per tank (g)	1,530	1,530	11,113
FCR	1.84	1.89	2.16

**TABLE 3:** Summary of means for BW and BW × SR for T1 harvested samples.

Tank	*N*	BW, Mean	BW, STDEV	BW, Rank, Low to High	BW × SR, Mean	BW × SR, STDEV	BW × SR, Rank, Low to High
RAS1	107	0.9274	0.74479	4	0.6581	0.52789	4
RAS2	118	0.6965	0.48101	1	0.5497	0.38011	1
RAS3	68	1.1338	0.95002	5	0.8322	0.69816	6
IPT1	93	1.2394	0.77299	6	0.7662	0.47816	5
IPT2	119	0.7087	0.50243	2	0.5608	0.39743	2
IPT3	101	0.8716	0.66915	3	0.5864	0.45050	3

**TABLE 4:** SGR at harvest of T2 and T3 post nursery culture trials.

Tank(s)	Input mass (g)	Final mass (g)	Mass gain (g)	SGR (%) d^−1^ (60 days)
IPS1	405	827	422	1.23
IPS2	317	1,158	841	2.23
IPS3	306	1,229	923	2.40
IPS T2 group	1,028	3,214	2,186	1.90
RAS1	401	1,007	606	1.59
RAS2	379	1,220	841	2.02
RAS3	361	971	610	1.71
RAS T2 group	1,141	3,198	2,057	1.71
IPS_large_	2,263	6,866	4,603	1.91
ALL	4,432	13,278	8,846	1.89

Notes: Group result is italicised.
